# The Effects of Intelectin-1 on Antioxidant and Angiogenesis in HUVECs Exposed to Oxygen Glucose Deprivation

**DOI:** 10.3389/fneur.2019.00383

**Published:** 2019-04-16

**Authors:** Naibing Gu, Jun Wang, Zhengli Di, Zhiqin Liu, Xiaotao Jia, Yu'e Yan, Xiaoshan Chen, Quanzeng Zhang, Yihua Qian

**Affiliations:** ^1^Department of Human Anatomy, Histology and Embryology, School of Basic Medical Sciences, Xi'an Jiaotong University Health Science Center, Xi'an, China; ^2^Department of Neurology, Xi'an Central Hospital, Xi'an Jiaotong University School of Medicine, Xi'an, China

**Keywords:** intelectin-1, OGD, HUVECs, angiogenesis, apoptosis, oxidative stress, PI3K/Akt

## Abstract

**Objective:** Ischemic stroke leads to cellular death and tissue damage by depriving the areas of glucose and oxygen supplies. The effective treatment of stroke remains a challenge for modern medicine. This study used an oxygen-glucose deprivation (OGD) model of human umbilical vein endothelial cells (HUVECs) to mimic ischemic injuries and explored the role and mechanism of intelectin-1.

**Methods:** Intelectin-1 was transduced into the HUVECs using a lentiviral vector. The PI3K/Akt signaling was examined in intelectin-induced eNOS phosphorylation. The PI3K inhibitor LY294002 was dealed in HUVECs.

**Results:** Our results demonstrated an increase in capillary density, decrease in apoptotic cells, and increase in HIF-1α protein expression following intelectin-1 treatment. Real-time PCR and Western blotting revealed the increased intelectin-1 expression alongside eNOS and Akt phosphorylation with enhanced bcl-2 expression under OGD. Capillary density decreased significantly after LY294002 treatment.

**Conclusion:** These results suggest intelectin-1 promotes angiogenesis, inhibits oxidative stress and reduces apoptosis by stimulating the Akt-eNOS signaling pathway in response to ischemia *in vitro*.

## Introduction

Stroke is a main reason of human neurological disability, ischemic stroke (IS) accounts for almost 80–90% of all strokes. IS occurs after a cerebral blood flow disruption, leading to cellular death and tissue damage by restricting glucose and oxygen supplies (1). Ischemic vascular diseases cause substantial vascular valve and vascular endothelial cell injuries, eventually damaging the surrounding tissues (2, 3).

Because of the complexity of acute IS pathophysiology, there are no effective methods and measures to treat acute IS. Previous research has focused on the neuronal and astrocytic damage following IS; however, as strokes also affect microvessels, vascular endothelial cell changes. Recent stroke research has paid great attention to the importance of neurovascular units (NVUs, is made up of neurons, endothelial cells, and astrocytes), because stroke affects neurons, astrocytes and microvessels at same time (4, 5). Within NVUs, endothelial cells are critical for the blood flow, oxygen and glucose delivery, and the regulation of cerebral microcirculation (6, 7). Vascular damage during ischemia often leads to disruption of the blood–brain barrier (BBB) and dysregulation of vascular tonus, eventually causing substantial cell death (8).

Induction of vascular regeneration after cerebral ischemia is one of the effective therapeutic strategies to promote post-stroke recovery (9). Endogenous neurogenesis and angiogenesis promote the formation of neuroblasts associated with vascular endothelial cells following IS (10–12). Angiogenesis leads to the recovery of cerebral blood flow at the ischemic penumbra in the central nervous system, contributing to long-term functional recovery from IS (13).

Akt and its downstream target eNOS are well-established crucial regulators of blood vessel growth and vascular cell function (14, 15). Intelectin-1 promotes vascular endothelial cell differentiation and survival by activating the Akt-eNOS signaling pathway *in vitro* (16). Recent study has confirmed that the anti-apoptosis and angiogenic effects of intelectin-1 are mediated by Akt-enos signal in rats with cerebral ischemia (17).

Lectins, a large family of calcium-dependent galactose-binding lectins, are conserved during evolution and are associated with many biological functions, including cell proliferation regulation, tumor antigen recognition and innate recognition of carbohydrates in cell walls of pathogens (9, 18, 19). In the previous study, We found that intelectin-1 could enhance the function and vascularization of vascular endothelial cells in ischemic rats, and inhibit the apoptosis of ischemic rats (17). However, Menzel et al. (20) observed that higher levels of omentin-1(intelectin-1) were significantly associated with a higher risk of stroke in metabolically healthy participants. In some tumor studies, scholars found that Intelectin-1 had the effect of inhibiting cell differentiation (21, 22). As seems to be contrary to our results. To further clarify the role of intelectin-1 in enhancing angiogenesis in an ischemic environment, human umbilical vein endothelial cells (HUVECs)-oxygen-glucose deprivation (OGD) model were used to investigate the effect and mechanism of intelectin-1 on mimic ischemic injury. We focused on whether intelectin-1 promotes endothelial cell function, angiogenesis and reduces apoptosis through PI3k / Akt signal pathway.

## Materials and Methods

### Cell Culture and Lentivirus Transduction

HUVECs (Geneticell Bioengineering Inc., Shanghai) were suspended in high glucose DMEM supplemented with 10% fetal bovine serum (Gibco). All cultures were maintained at 37°C under an atmosphere containing 95% O_2_ and 5% CO_2_. Cells from passages 3–5 were used for experiments. Lentivirus vector-mediated intelectin-1(LV-I1) or control (LV-C) conjugated with green fluorescent protein (GFP) were transduced into HUVECs. Forty eight hours of after transduction, HUVEC imaging taken by OLYMUPS BX51 fluorescence microscope was used to analyze the transduction efficacy of LV-I1. The HUVEC passaged after transduction was used for the following groups of tests: normoxia; OGD; OGD treated with LV-C; OGD treated with LV-I1 and OGD treated with LV-I1+ LY294002.

### Oxygen Glucose Deprivation and Morphology

After HUVECs were cultured for 24 h, it was replaced with glucose-free DMEM and OGD was achieved using the disclosed method (23). The LY294002 group was further treated with glucose-free DMEM containing LY294002 (50 mol/liter) for 30 min. The HUVECs were then cultured in an anaerobic chamber containing 5% CO_2_ and 95% N_2_ (v / v) for 6 h in a humidified incubator at 37 °C. The OGD condition was terminated by replacing the medium with normal medium before putting back to normoxic culture conditions. Normal control cells were cultured in the same experimental procedure, but were not exposed glucose-free DMEM or anoxia. Morphological changes after OGD were observed under an inverted Eclipse TE200 (Nikon, Japan) microscope.

### MTT Assay

Cellular viability was assessed using a mitochondrial assay kit according to the manufacturer instructions: 10 μL MTT labeling reagent at a final concentration of 0.5 mg/mL was added into each well at the termination of OGD and before incubation, which occurred in a humidified incubator at 37°C with 5% CO_2_ and 95% air (v/v) at 90% humidity for 4 h to allow purple formazan crystal formation. Four hours later, 100 μL of the solubilization reagent was added into each well. Finally, the solubilized purple formazan crystals were measured using a microplate reader at an absorbance wavelength of 570 nm. All MTT results were normalized and expressed as percentages of the average optical density reading from the normal control group.

### Tube Formation

A 24-well culture plate was precoated with 250 μL growth factor-reduced Matrigel (Sigma, USA) at 37°C for 30 min. Six hours after OGD, 4 × 10^4^ cells (in 300 μL of DMEM) from each group were seeded onto the Matrigel-coated plates. After 4 h, the capillary structure formation was examined under an optical microscope. The formation of vascular-like structures was evaluated according to the instructions (24). Network formation was observed under an inverted phase contrast microscope (Nikon,Tokyo, Japan). The degree of tube formation was evaluated by measuring the length of tubes in three randomly chosen fields from each well with the Image J software. Each experiment was repeated three times.

### The Expression of Intelectin-1 mRNA Assays by Quantitative Real-Time PCR (qRT-PCR)

All HUVEC RNA (normoxia, *n* = 3; OGD, *n* = 3; LV-C, *n* = 3; LV-I1, *n* = 3; LY294002, *n* = 3) was extracted with TRIzol (Invitrogen Life Technologies, Carlsbad, CA, USA) 6 h after OGD. First-strand cDNA was made with PrimeScript RT reagent kit (Takara, Tokyo, Japan). SYBR Green (Perfect Real-Time Kit (Takara) real-time PCR was carried out by intelectin-1 forward primer: 5-TGACAATGGTCCAGCATTACC-3, and reverse primer: 5- ACGGGGTTACCTTCTGGGA-3. in a Rotor-Gene 3000 system (Corbett Research, Sydney, Australia). The PCR amplification conditions were: initial denature at 95°C for 10 s followed by 40 cycles of at 95°C for 5 s, 60°C for 20 s. The cycle threshold (Ct) of each gene transcript was normalized against that of β-actin. Fold changes were determined using 2^−ΔΔ*CT*^ method.

### The Proteins of Intelectin-1, Bcl-2, HIF-1a, Akt, p-Akt, eNOS, and p-eNOS Analysis by Western Blotting

Six hours after OGD, the HUVECs were washed twice with cold PBS. The proteins were prepared with protein extraction reagent containing phenylmethylsulfonyl fluoride (PMSF) (Pierce Biotechnology Inc., Rockford, IL, USA). The protein concentration was determined by BCA protein assay reagents (25). The samples (20 μg of protein) were electrophoresed on 10% SDS–PAGE, subsequently transferred onto a nitrocellulose membrane. The membranes were incubated with 10% skim milk in Tris-buffered saline with 0.05% Tween-20 (TBST) for 1 h at room temperature. The membranes were then incubated with primary antibodies (anti–intelectin-1, 1:200; anti-Bcl-2, 1:1000; anti-HIF-1a antibody, 1:500; anti-Akt, p-Akt, eNOS, p-eNOS, 1:200; and anti β-actin,1:5000) for 12–16 h at 4°C. After that, the membranes were washed with TBST, and incubated with secondary antibodies conjugated with horseradish peroxidase at 1:1000. The immunoreactive bands were observed using SuperSignal West Pico chemiluminescent substrate, an enhanced chemiluminescence kit, according to the instruction of technical manual, and densitometry was performed with Image J.

### Detection of Superoxide Dismutase Activity

Superoxide dismutase (SOD) activity was measured using WST according to published protocol (26).

### Intracellular Level of Reactive Oxygen Species (ROS)

Cellular oxidative stress was detected by the reactive oxygen species (ROS)-mediated conversion of 2′,7′-dichlorofluorescein diacetate (DCF-DA) into fluorescent DCF. This allows cellular oxidation to be measured in viable cells (27). 6 h after OGD treatment, the cultured HUVECs were incubated in 100 μM DCF-DA (Molecular Probes, Leiden, Netherlands) for 50 min, followed by HBSS washes three times. A fluorescence microplate reader (Fluoroscan, Labsystems, Finland) was used to detected DCF fluorescence. The excitation wavelength of 485 nm was filtered using a 538 nm-barrier filter. Autofluorescence of cells which were not loaded with DCF-DA corrected all fluorescent measurements (a constant value throughout the experiment). Level of reactive oxygen species was expressed as a percentage of the control (normoxia).

### TUNEL Assay

The HUVECs plated on 24-well chamber slides (Millipore, Billerica, MA) were subjected to OGD treatments as described previously: cells were fixed in 4% paraformaldehyde for 25 min, then treated with permeabilization solution (0.2% Triton X-100 solution in PBS) for 5 min at room temperature, followed by PBS washes three times for 5 min. The slices were then incubated in medium containing an endogenous peroxidase blocker (0.3% H_2_O_2_ solubilized in methanol) for 30 min at room temperature and the PBS washes were repeated. Subsequently, 50 mL TUNEL reagent was added onto the slices and cultivated in a humidified chamber at 37°C for 60 min, then washed with PBS. The slices were then incubated in 50 mL transforming agent-peroxidase in similar conditions for 30 min and re-washed with PBS. One hundred of milliliters DAB was used to stained the slices at room temperature, Subsequently counterstained with hematoxylin. After the slides were washed, dehydrated, cleaned, etc., fixed for observation under a light microscope.

Normal cell nuclei were stained blue, whereas apoptotic cell nuclei were stained hyperchromically. Apoptotic cells were characterized by dark-brown nuclei, with visible chromosomal condensation. Five fields were selected for each slice, the numbers of positive cells were taken, and the average was calculated.

### Data Analysis

SPSS17.0 software (SPSS, Chicago, IL, USA) was used to analyzed data presented in mean ± SEM. One-way ANOVA were used to assessed the statistical differences among the groups, followed by an SNK test. *P* < 0.05 was considered statistically significant.

## Results

### Widely Expressed Green Fluorescence and Significantly Upregulated Intelectin-1 in HUVECs

**Figure 1:**
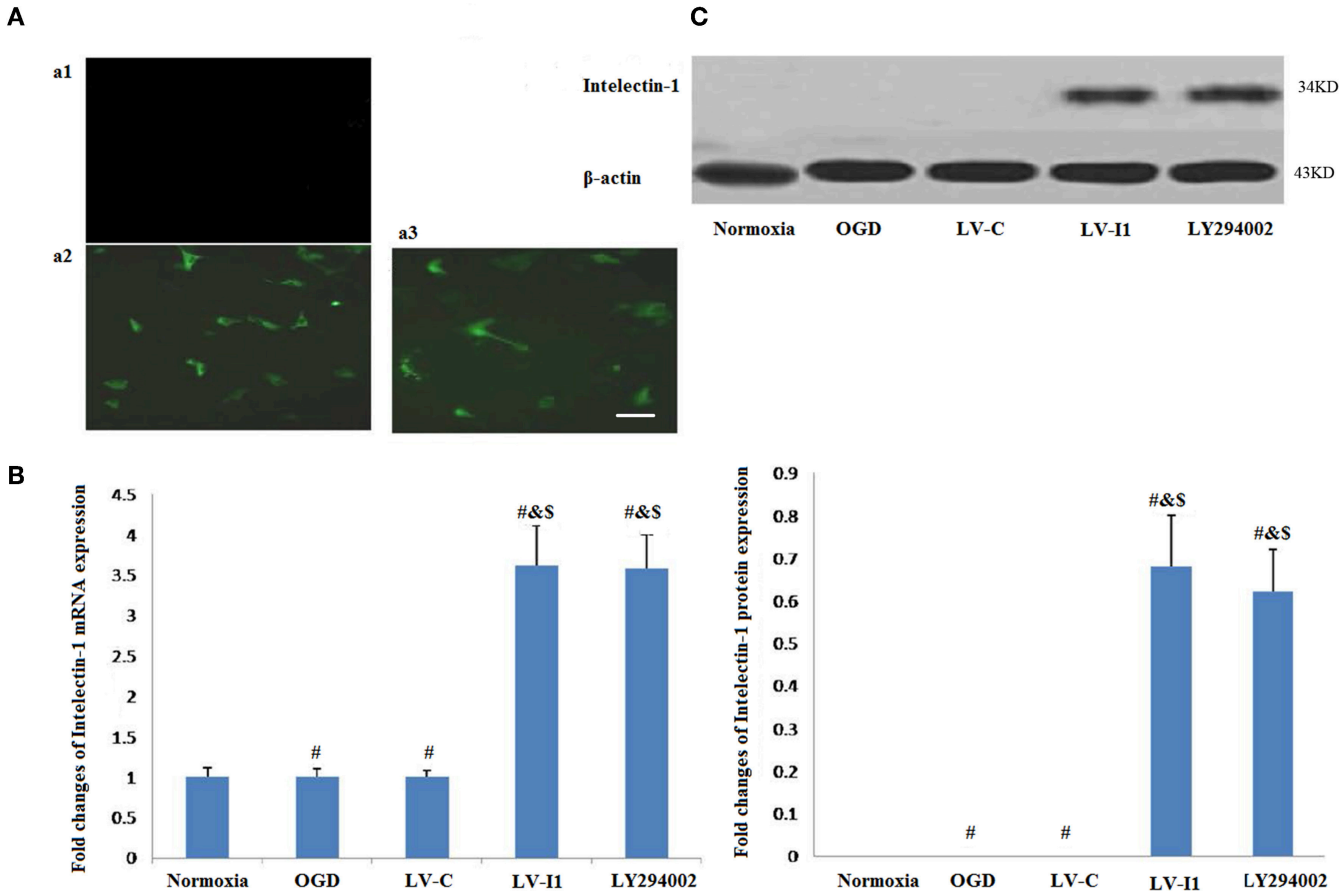
LV-I1 transduction increased the expression of intelectin-1 mRNA and protein in HUVECs of OGD. The results were expressed as mean ± SEM in each group (*n* = 3). **(A)** The fluorescence was not observed in normal HUVECs without transduction (a1). Green fluorescence was widely expressed in HUVECs transduced by LV-C and LV-I1, respectively (a2 and a3). Scale bar = 50 μm. **(B)** Expression of intelectin-1 mRNA in HUVECs exposed to OGD. Intelectin mRNA is almost few expressed in the normoxia, OGD and LV-C groups. However, the intelectin-1 mRNA level in LV-I1 and LY294002 groups were higher significantly than in the other three groups (^#^*P* < 0.01, vs. Normoxia; ^&^*P* < 0.01, vs. OGD group; ^*$*^*P* < 0.01, vs. LV-C group). There was not significant difference between LV-I1 and LY294002 groups. **(C)** Expression of intelectin-1 protein in HUVECs exposed to OGD. Intelectin protein is almost few expressed in the normoxia, OGD and LV-C groups. However, the intelectin-1 protein level in LV-I1 and LY294002 groups were higher significantly than in the other three groups (^#^*P* < 0.01, vs. Normoxia; ^&^*P* < 0.01, vs. OGD group; ^*$*^*P* < 0.01, vs. LV-C group). There was not significant difference between LV-I1 and LY294002 groups.

Forty hours after transduction, there was no fluorescence observed in normal HUVECs (Figure 1Aa1), but it was observed in LV-C and LV-I1 transduced HUVECs under a fluorescence microscope (Figures 1A,a2,3), suggesting that LV-C and LV-I1was successfully transduced into HUVECs.

In order to evaluate LV-I1 transduction efficiency in HUVECs, intelectin-1 mRNA and protein expression levels were determined using qRT-PCR and Western blotting after OGD. Intelectin-1 was not expressed in the normoxia, OGD, and LV-C groups; but intelectin-1 protein and mRNA expression levels of were notably higher in the LV-I1 and LY294002 groups (*P* < 0.01; Figures 1B,C). There was no significant difference between the LV-I1 and LY294002 groups (*P* >0.05; Figures 1B,C), indicating that expression level of intelectin-1 was obviously increased after LV-I1 administration in HUVECs undergoing OGD.

### Cell Morphology

**Figure 2:**
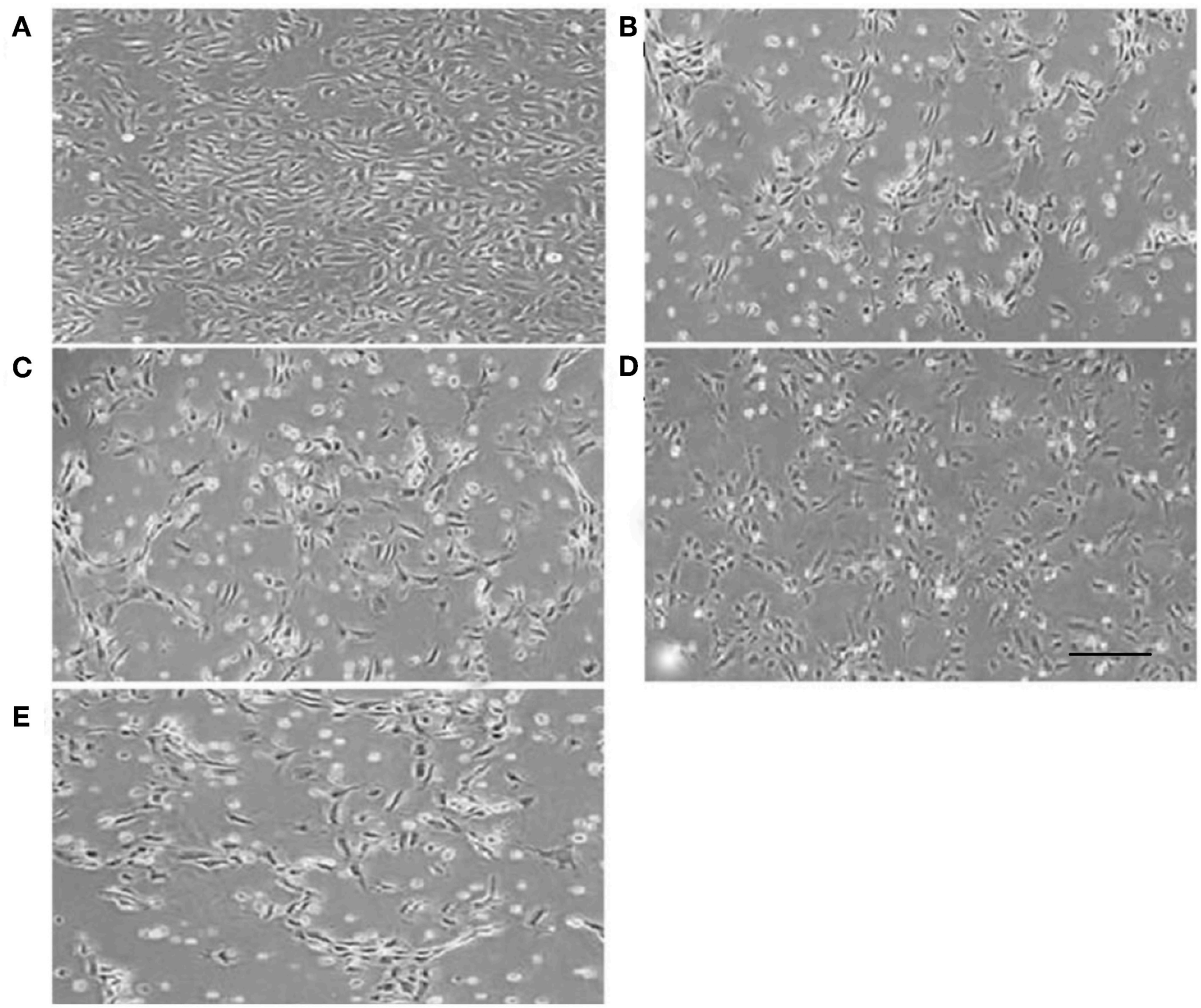
Human umbilical vein endothelial cell morphology. Human umbilical vein endothelial cells were flat polygonal or fusiform, paving stone-like growth under normoxic conditions **(A)**. After OGD, HUVECs became star-shaped and totally detached from each other **(B,C,E)**. Human umbilical vein endothelial cells became polygonal or fusiform after intervention with LV-I1 **(D)**. Scale bar = 50 μm.

Normal HUVECs displayed flat polygonal or fusiform, paving stone-like growth (Figure 2A); however, cells that underwent 6 h OGD treatment exhibited dramatic morphology changes, becoming elongated and starting to widen each other. Some cells in the OGD and LV-C groups tended to become star-shaped to the point of completely detaching from the dish (Figures 2B,C). After LV-I1 treatment, intelectin-1 effectively prevented these OGD-induced morphological changes in endothelial cells (Figure 2D). After the pI3K pathway was blocked, this star-shaped cell morphology trend was significantly enhanced (Figure 2E).

### Intelectin-1 Reduced OGD-Induced Cell Damage

**Figure 3:**
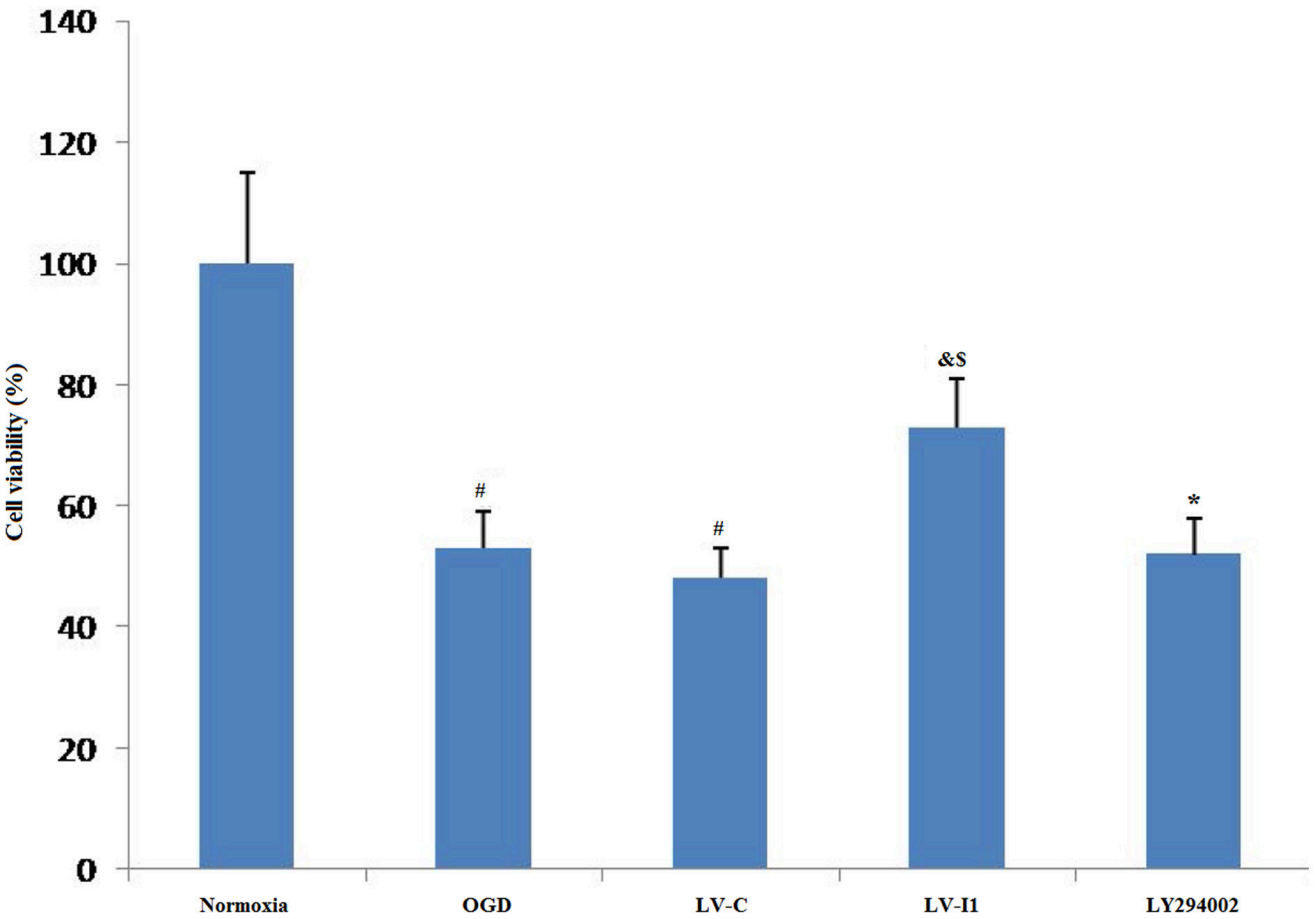
Intelectin-1 reduced OGD-induced cell damage. The cell viability was evaluated by MTT assay. The human umbilical vein endothelial cell viability in OGD and LV-C groups was significantly lower than that in the normoxia group (^#^*P* < 0.05, vs. normoxia group). However, there was not significant difference between OGD and LV-C groups. After intelectin-1 treatment, the cell viability was increased obviously in the LV-I1 group (^&^*P* < 0.05, vs. OGD group; ^*$*^*P* < 0.05, vs. LV-C group). The cell viability of LY294002 group was decreased significantly (^*^P < 0.05, vs. LV-I1 group).

The relative MTT values after 6 h OGD were ~53 and 48% in OGD and LV-C groups, respectively; but no significant changes were noted between them (*P* > 0.05; Figure 3). After intelectin-1 intervention, MTT value was ~73% in LV-I1 group (*p* < 0.05; Figure 3), indicating that intelectin-1 can protect HUVECs against OGD insult.

After intervention with the PI3k pathway inhibitor LY294002, MTT value in LY294002 group was ~52% (*p* < 0.05; Figure 3), indicating that intelectin-1 may play a protective role in cell activity through the PI3k pathway.

### *In vitro* Intelectin-1 in HUVECs Induced Tube Formation After Hypoxia

**Figure 4:**
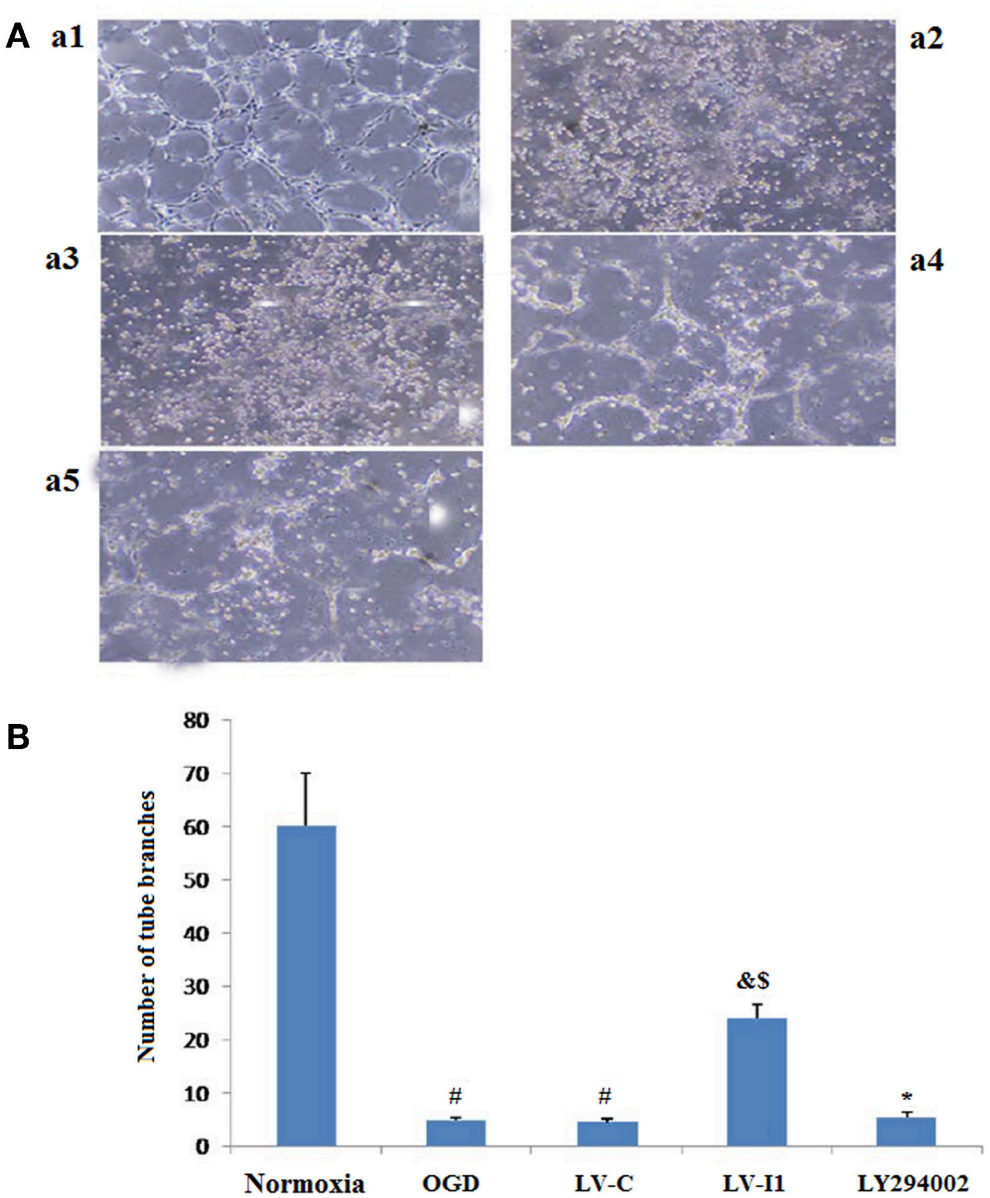
Intelectin-1 in HUVECs induced tube formation. The results were expressed as mean ± SEM in each group (*n* = 3). Compared with the normoxia group (a1), the number of tubes in OGD (a2) and LV-C (a3) groups was reduced significantly (^#^*P* < 0.05, vs. normoxia group). The number of tubes in the LV-I1 group (a4) was increased obviously compared with the OGD and LV-C groups (^&^*P* < 0.05, vs. OGD group; ^*$*^*P* < 0.05, vs. LV-C group). The number of tubes decreased significantly in the LY294002 group (a5) (^*^P < 0.05, vs. LV-I1 group). **(B):** Tube formation numbers.

After HUVECs exposed to oxygen glucose deprivation (OGD) for 6 h, cellular tube formation was investigated. OGD and LV-C group microtubule formation was significantly reduced compared to the normoxia group (*P* < 0.05; Figures 4A,B), but no significant differences were noted between the OGD and LV-C (*P* > 0.05; Figures 4A,B).Treatment with LV-I1 increased the number of formed tubes significantly compared to the OGD or LV-C groups (*P* < 0.05; Figures 4A,B). After use of LY294002, the tubes number decreased significantly (*P* < 0.05; Figures 4A,B). *In vitro* administration of LV-I1 may thus enhance angiogenesis through the PI3k pathway following OGD.

### Intelectin-1 Decreased Cellular Apoptosis in HUVECs

**Figure 5:**
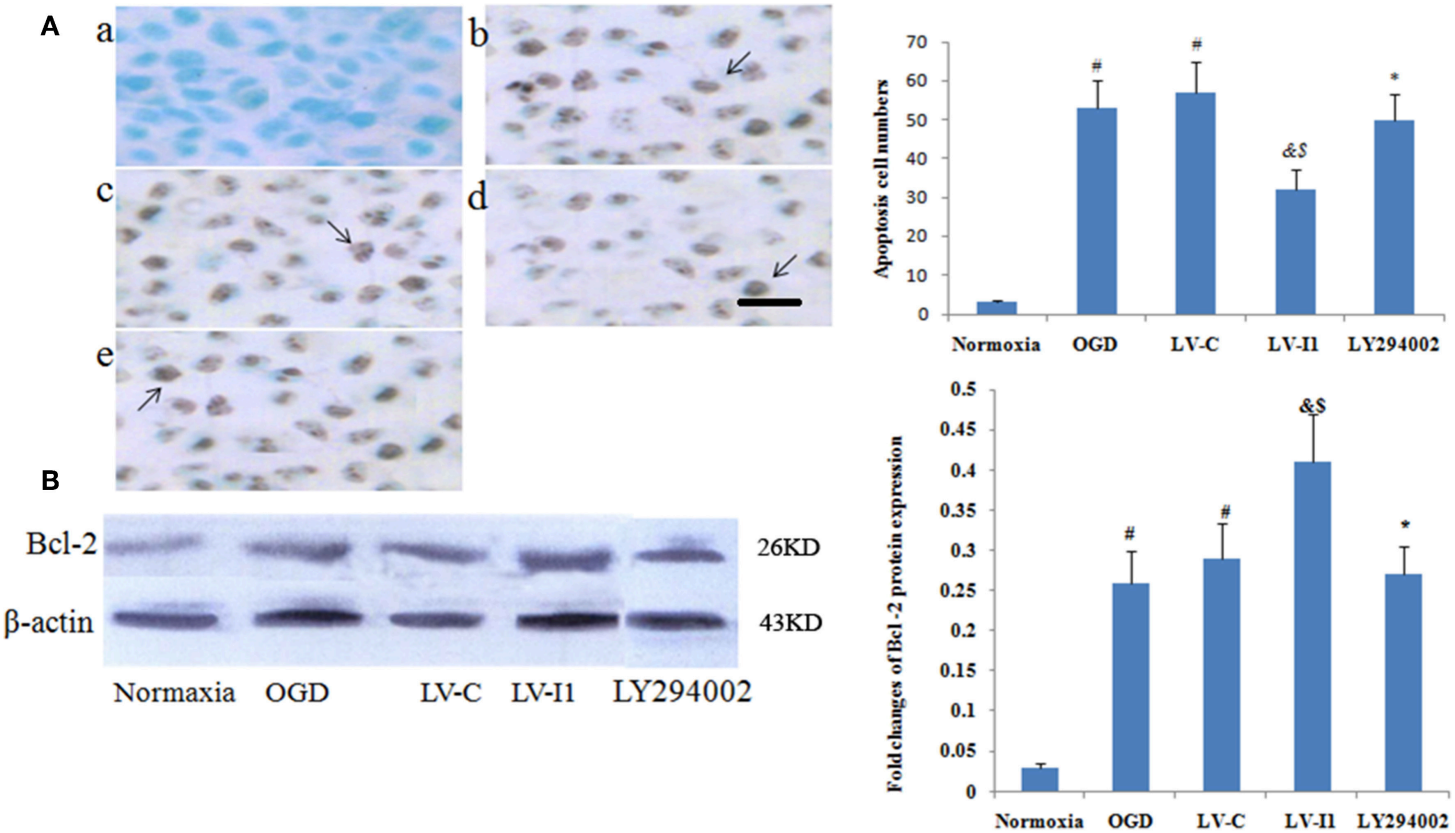
Intelectin-1 reduced cellular apoptosis in HUVECs. The results were expressed as mean ± SEM in each group (*n* = 3). **(A)** The number of apoptotic cells (TUNEL method) in the HUVECs exposed to OGD. The number of apoptotic cells in the OGD (b) and LV-C (c) groups were higher significantly than in the normoxia group (a) (^#^*P* < 0.05, vs. normoxia group). The number of apoptotic cells was decreased significantly in the LV-I1 group (d) (^&^*P* < 0.05, vs. OGD group; ^*$*^*P* < 0.05, vs. LV-C group). Following intervention with LY294002, the apoptotic cells in the LY294002 group (e) were increased significantly compared with the LV-I1 group (^*^P < 0.05, vs. LV-I1 group). Scale bar = 50 μm. **(B)** Expression of Bcl-2 protein in HUVECs exposed to OGD. The Bcl-2 protein level was increased markedly in OGD and +LV-C groups compared with the level in the normoxia group (^#^*P* < 0.05, vs. normoxia group). The expression level of Bcl-2 was further upregulated significantly in the LV-I1 group (^&^*P* < 0.05, vs. OGD group; ^*$*^*P* < 0.05, vs. LV-C group). The expression level of Bcl-2 was decreased significantly in the LY294002 group compared with the level in the LV-I1 group (^*^P < 0.05, vs. LV-I1 group).

Apoptotic cells were detected among OGD, LV-C, LV-I1 and LY294002 group HUVECs characterized by dark-brown nuclei (Figures 5A,b–e). The number of HUVEC apoptosis was markedly higher in the OGD and LV-C groups than in normoxia group (*P* < 0.05; Figure 5A). After LV-I1 treatment, the apoptotic cells number decreased significantly (*P* < 0.05; Figure 5A). After LY294002 intervention, the LY294002 group displayed significantly more apoptotic cells than that of the LV-I1 group (*P*<*0.05*; Figure 5A). The above results indicate that intelectin-1 inhibited apoptosis via PI3k pathway.

Furthermore, Bcl-2 expression was analyzed via Western blotting 6 h after OGD. protein levels of Bcl-2 were obviously increased in the OGD and LV-C groups *(P* < 0.05; Figure 5B). After LV-I1, Bcl-2 level was markedly further increased in this group (*P* < 0.05; Figure 5B), indicating that intelectin-1 reduced apoptosis by adding Bcl-2 expression. After PI3k pathway inhibitor LY294002 intervention in the LY294002 group, the Bcl-2 expression level decreased significantly (*P* < 0.05; Figure 5B).

### Intelectin-1 Enhanced OGD-Induced HIF-1α Expression

**Figure 6:**
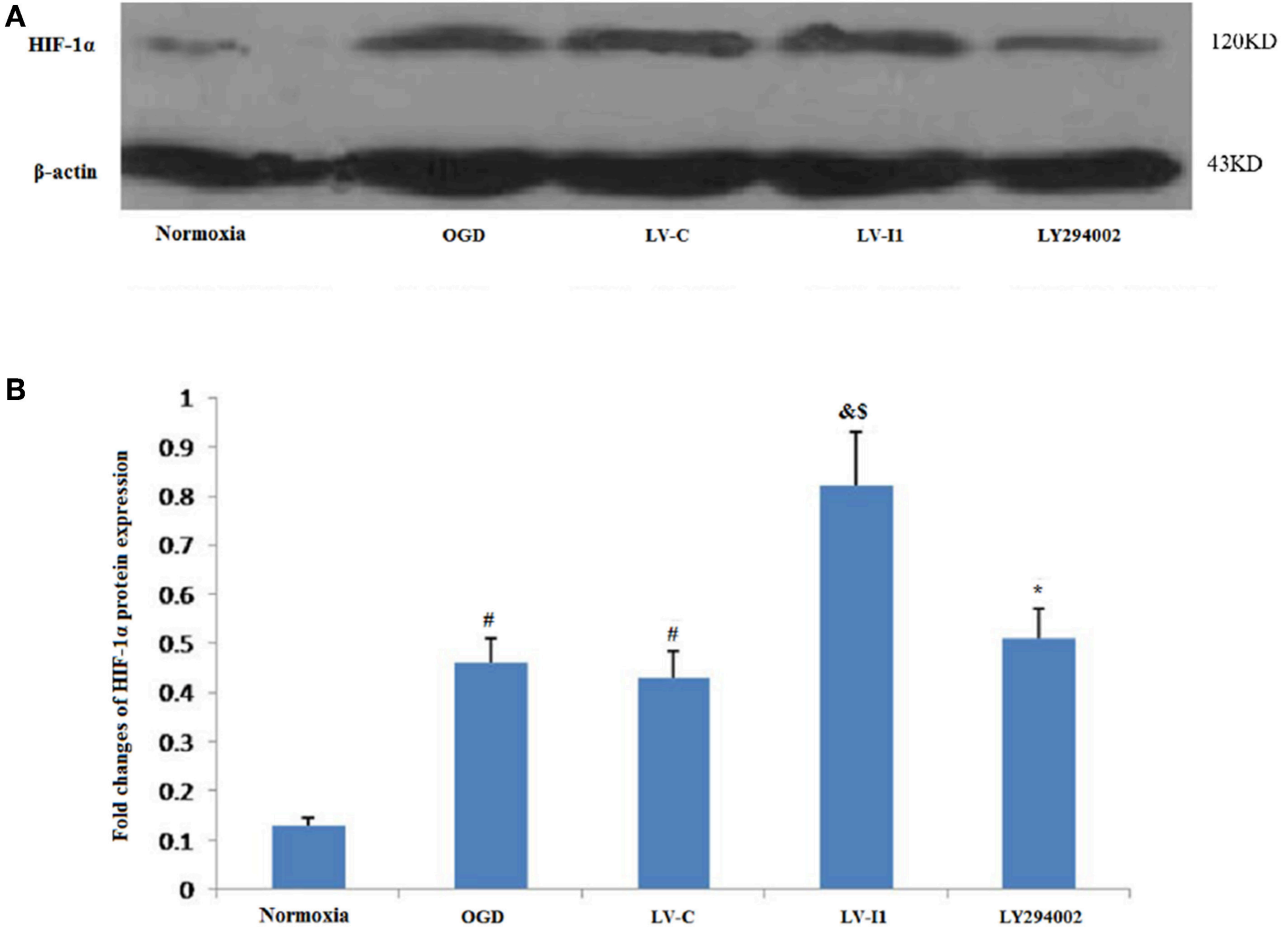
Intelectin-1 enhanced OGD-induced HIF-1α expression. The results were expressed as mean ± SEM in each group (*n* = 3). **(A)** Expression of HIF-1α protein in HUVECs exposed to OGD. **(B)** HIF-1α is almost few expressed in the normoxia group. However, HIF-1α protein level in the OGD and LV-C groups were higher significantly than in the normoxia group (^#^*P* < 0.05, vs. normoxia group). There was not significant difference between OGD and LV-C groups. The expression level of HIF-1α was further upregulated significantly in the LV-I1 group (^&^*P* < 0.05, vs. OGD group; ^*$*^*P* < 0.05, vs. LV-C group). The expression level of HIF-1α was decreased significantly in the LY294002 group compared with the level in the LV-I1 group (^*^P < 0.05, vs. LV-I1 group).

Almost hypoxia inducible factor-1alpha (HIF-1α) was not measured in HUVECs under normal conditions. Following 6 h OGD exposure, however, Protein level of HIF-1α expression was measured in the OGD and LV-C groups (*P*<*0.05*; Figure 6A). HIF-1α expression was greater than with OGD (*P* < 0.05) after LV-I1 treatment, though after the PI3k signal way was inhibited, HIF-1α expression was notably reduced in the LY294002 group. (*P* < 0.05; Figure 6B). These results suggested that intelectin-1 increased HIF-1α expression.

### Intelectin-1 Attenuated Cellular Oxidative Stress in HUVECs Exposed to OGD

**Figure 7:**
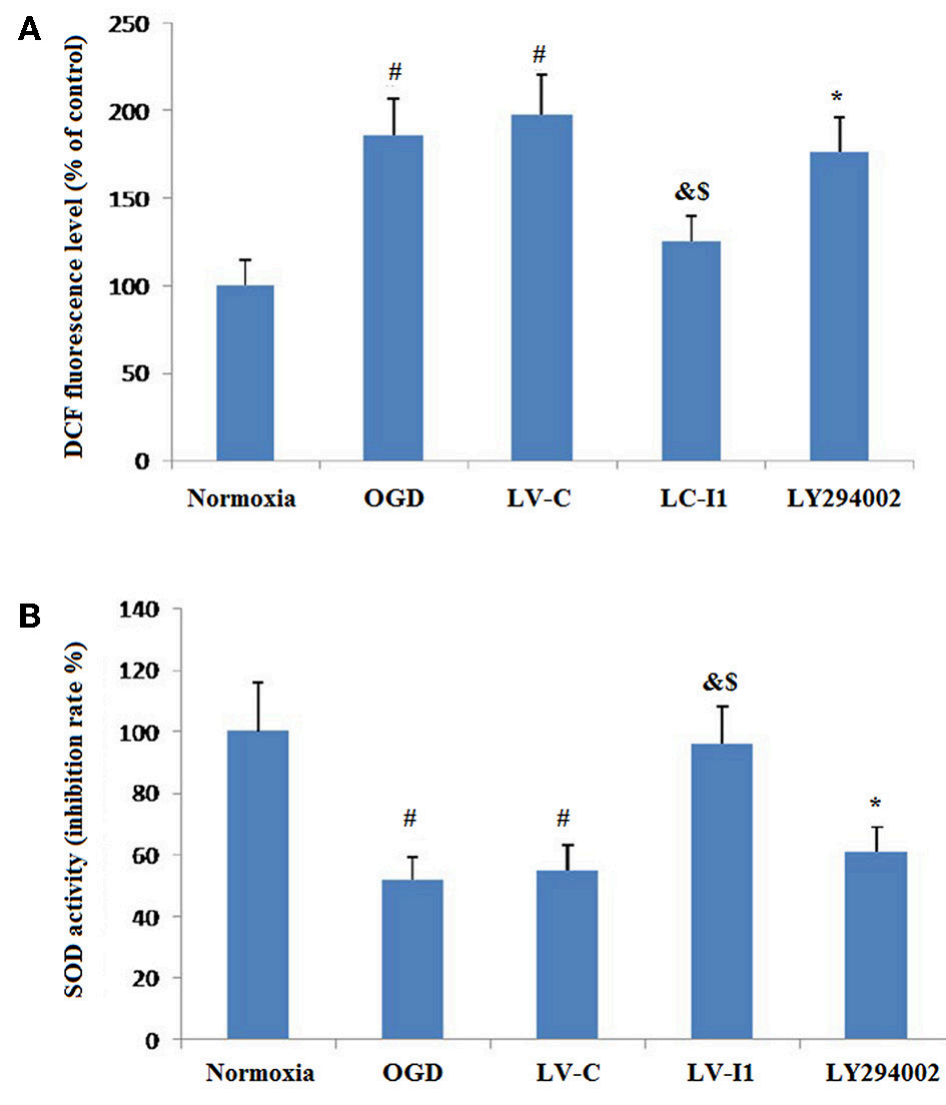
Intelectin-1 attenuated cellular oxidative stress in HUVECs exposed to OGD. The results were expressed as mean ± SEM in each group (*n* = 3). **(A)** ROS level in HUVECs exposed to OGD. DCF fluorescence level in the OGD and LV-C groups were higher significantly than in the normoxia group (^#^*P* < 0.05, vs. normoxia group). The level of DCF fluorescence was downregulated significantly in the LV-I1 group (^&^*P* < 0.05, vs. OGD group; ^*$*^*P* < 0.05, vs. LV-C group). The level of DCF fluorescence was increased significantly in the LY294002 group compared with the level in the LV-I1 group (^*^P < 0.05, vs. LV-I1 group). **(B)** SOD activity in HUVECs exposed to OGD. SOD activity level in the OGD and LV-C groups were lower significantly than in the normoxia group (^#^*P* < 0.05, vs. normoxia group). The level of SOD activity was upregulated significantly in the LV-I1 group (^&^*P* < 0.05, vs. OGD group; ^*$*^*P* < 0.05, vs. LV-C group). The level of SOD activity was decreased significantly in the LY294002 group compared with the level in the LV-I1 group (^*^P < 0.05, vs. LV-I1 group).

Figure 7 showed the effects of intelctin-1 on cellular oxidative stress. DCF fluorescence increased considerably with simulated ischemia *in vitro* (*P* < 0.05), suggesting that OGD increased ROS expressive level in HUVECs. Figure 7A showed that a significant decrease of DCF fluorescence was measured in LV-I1 group (*P* < 0.05), suggesting that the oxidative stress of the cells is significantly weakened, while the treatment with LY294002 significantly increased the fluorescence of DCF (*P* < 0.05).

Superoxide dismutase is a vital enzymatic elements by which superoxide anions are eliminated. To investigate the possibility of intelectin-1influences on OGD-induced decreases in Superoxide dismutase activity, superoxide dismutase activity in cell cultures was assessed after oxygen glucose deprivation. The Superoxide dismutase activities of the normoxia, OGD, LV-C, LV-I1, and LY294002 groups were 100, 55.12, 55.56, 96.26, and 61.12% respectively (Figure 7B). Contrast with normoxia, Superoxide dismutase markedly inhibited SOD activity, an effect that was notably attenuated by intelctin-1 pretreatment (*P* < 0.05; Figure 7B). After LY294002 treatment, however, SOD activity decreased significantly (*p* < 0.05; Figure 7B).

### Intelectin-1 Stimulated Phosphorylation Level of Akt and eNOS

**Figure 8:**
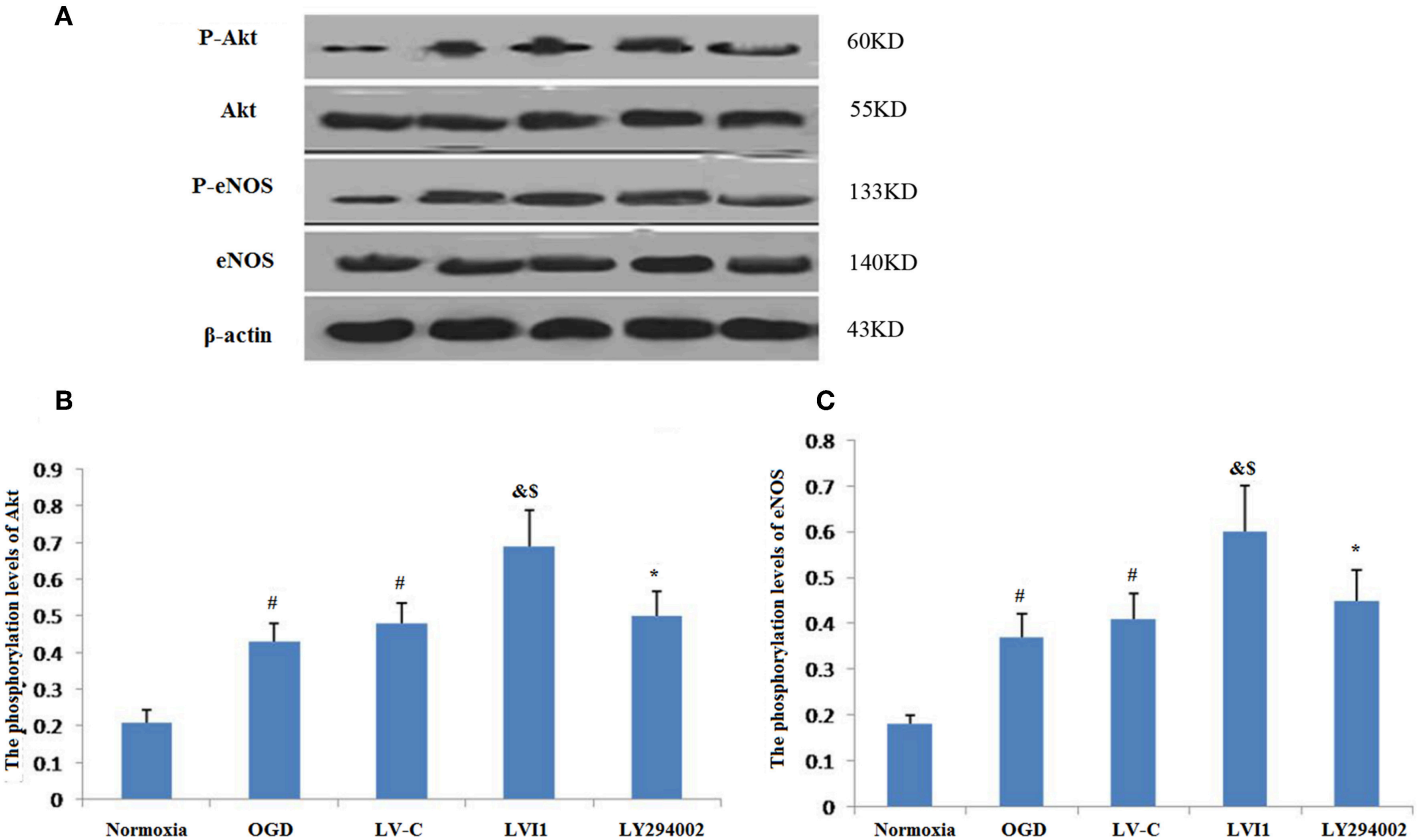
Intelectin-1 stimulated the phosphorylation of Akt and eNOS **(A)**. The results were expressed as mean ± SEM in each group (*n* = 3). **(B,C)** The phosphorylation levels of Akt and eNOS levels from the OGD and LV-C groups were increased significantly compared with the normoxia group (^#^*P* < 0.05, vs. normoxia group). However, there was no marked difference between the OGD and LV-C groups on the phosphorylation levels of Akt and eNOS. The phosphorylation levels of Akt and eNOS were increased substantially in the LV-I1 group than in the OGD and LV-C groups group (^&^*P* < 0.05, vs. OGD group; ^*$*^*P* < 0.05, vs. LV-Con group). After intervention with PI3k pathway inhibitors LY294002, the phosphorylation levels of Akt and eNOS were decreased substantially in the LY294002 group (^*^P < 0.05, vs. LV-I1 group).

Intelectin-1 treatment promoted Akt phosphorylation, which has a vital effect on the angiogenesis in endothelial cells (14). Since Akt phosphorylation promotes eNOS activities at Ser-1177 (15, 28), eNOS phosphorylation levels were also measured. Intelectin-1 promoted eNOS phosphorylation in a manner same as previous reported results, suggesting that intelectin-1 promoted phosphorylation of eNOS (29). As PI3K plays a role upstream in the Akt-eNOS signal way after activated by multi-factors (14). Phosphorylation levels of Akt and eNOS was observed to increased significantly in the OGD and LV-C groups compared to the normoxia group (*P* < 0.05; Figures 8A–C). No significant differences were observed in Akt and eNOS phosphorylation levels between the OGD and LV-C groups (*P* > 0.05; Figures 8A–C). In contrast, Akt and eNOS phosphorylation levels were obviously upregulated in the LV-I1 group compared to the OGD and LV-C groups (*P* < 0.05, Figures 8A–C). Furthermore, following intervention with PI3k pathway inhibitor LY294002, Akt and eNOS phosphorylation levels decreased notably in the LY294002 group (*P* < 0.05; Figures 8A–C). The above data indicated that intelectin-1 stimulated phosphorylation of Akt and eNOS, potentially promoting angiogenesis, inhibiting apoptosis, and attenuating oxidative stress by activating Akt-eNOS pathway in ischemic environment.

## Discussions

Previous studies have demonstrated intelectin-1 stimulates the Akt-eNOS signaling pathway, promotes endothelial cell function and revascularization, and inhibits apoptosis in ischemic responses (17). The OGD model is an important *in vitro* experimental tool for IS studies (30). Our study used OGD to simulate the HUVEC ischemic environment, studied the protective mechanism of intelectin-1 during IS, and verified intelectin-1 role in promoting angiogenesis and reducing apoptosis.

Firstly, lentiviral vectors (LVs) were used to deliver intelectin-1 to the HUVECs. After 48 h, Green fluorescent protein signal was detected in the HUVECs. Results further demonstrated that LV-I1 transduction increased protein and mRNA levels of intelectin-1by RT-PCR and Western blotting, revealing the intelectin-1 gene had been effectively transduced into the HUVECs with sustained expression. Secondly, the viability and morphology of HUVECs exposed to OGD were examined. It was found that cell viability under OGD decreased significantly in the OGD and LV-C groups, but increased notably after LV-I1 treatment, compared to the OGD group. At the same time, the morphology of the cells observed under OGD showed that after intelectin-1 intervention, that the cells changed from star-shapes to elongated-shapes, indicating that intelectin-1 enhances cell viability under hypoxic conditions.

The importance of NVUs is currently receiving significant concern in the field of stroke-researcher because stroke can affect neurons, microvessels and astrocytes (4). Brain microvascular endothelial cells are the most prominent cell type in NVUs (31), and are highly important for the reperfusion of blood after a cerebral infarction. The central role of endothelial cells in the pathobiology of cerebral blood vessels has been encompassed under the term NVUs (32). Increasing experimental evidence has shown that microvascular endothelial cells (BMECs) are not simply inert tubes for delivering blood, oxygen, and glucose to the brain, but that they also secrete directly neuro protective trophic factors (33). Similarly, this experiment found that intelectin-1 promoted the formation of tubes in HUVECs, a sign of angiogenesis after hypoxia. This is consistent with the previous reports that showed intelectin-1 activated cell differentiation and revascularization (16). Since LY294002 significantly reduced the number of tubes after treatment, Intelectin-1 promotes angiogenesis after OGD through the PI3k pathway. Apoptosis is an orderly process of energy-dependent programmed cell death (34). B-cell lymphoma-2 (Bcl-2) which belongs to an anti-apoptotic protein of the Bcl-2 family acts as a vital role in the cerebral ischemic injury (35–37). The data showed that the Bcl-2 protein expression and the apoptotic cells number were obviously elevated in HUVECs after OGD. After LV-I1 intervention, Bcl-2 expression was further increased; however, the apoptotic cells number in this group inhibited, demonstrating that intelectin-1 inhibited cell apoptosis by up-regulating B-cell lymphoma-2 expression.

HIF-1α is one of the master regulators regulating the cellular responses to hypoxia. Many studies have shown that elevated HIF-1α levels play protective roles in reducing cellular apoptosis in ischemic brain damage (38). By controlling the transcription of the hundreds of genes that HIF-1α drives, many cellular processes including angiogenesis may be activated (39). The HIF-1α protein is hardly expressed in most normoxic cells (40); however, under hypoxia, HIF-1α transcription is activated, up-regulating more HIF-1α protein, and oxygen-sensing mechanisms immediately stabilize these proteins (41, 42). Consistent with previous observations, this study showed that normal HUVECs expressed little HIF-1α protein, whereas HUVECs that underwent OGD expressed increased HIF-1α protein levels. Intelectin-1 further upregulated the level of HIF-1α protein expression in the HUVECs that underwent OGD. The upregulation of the HIF-1α protein expression was associated with protective effect of intelectin-1 during OGD.

Reactive oxide species contribute significantly to ischemic brain injury, making ROS suppression a protective mechanism. Under normal biological conditions, Reactive oxygen species production and clearance are balanced. Under OGD conditions, this balance is disrupted, resulting in excessive ROS. This study found that ROS increased significantly after hypoxia; however, pretreatment with intelectin-1 significantly reduced ROS levels.

Although hypoxic stress reduced SOD production, intelectin-1 significantly attenuated the OGD-induced down-regulation of superoxide dismutase activity. However, the effect caused by the direct action of intelectin-1 direct SOD or an indirect action through inhibiting mitochondrial superoxide activity are not yet known. Needless to say, this result further demonstrates the antioxidant effects of intelectin-1.

Our data indicated that Akt and eNOS phosphorylation levels were significantly upregulated after hypoxia. Following pretreatment with intelectin-1, these phosphorylation levels further increased. Under the same pretreatment, it was found that cellular tube formation and cellular activity increased significantly, whereas apoptosis and oxidative stress were significantly inhibited. After LY294002 intervention, cellular tube formation and cellular activity decreased significantly, alongside significant enhancements in apoptosis and oxidative stress. The above data indicate that neuroprotective activity of intelectin-1 may be through PI3K signal pathway to reduce apoptosis and promote angiogenesis.

## Conclusions

This research showed that intelectin-1 acted as a novel angiogenesis regulator, oxidative stress inhibitor, and anti-apoptotic agent in an OGD model using HUVECs. Treatment with LV-I1 in HUVECs exposed to OGD triggered angiogenesis, inhibited oxidative stress, and reduced apoptosis. After the intervention of PI3k pathway inhibitor LY294002, oxidative stress and apoptosis increased, and tube formation decreased. Therefore, anti-apoptotic, oxidative stress inhibition, and angiogenesis of intelectin-1 are mediated by stimulation of PI3K signaling stimulation.

## Author Contributions

NG and JW contributed to the conception of the study. ZD and ZL contributed significantly to analysis and manuscript preparation. XJ, YY, and XC performed the data analyses and wrote the manuscript. QZ and YQ helped perform the analysis with constructive discussions.

### Conflict of Interest Statement

The authors declare that the research was conducted in the absence of any commercial or financial relationships that could be construed as a potential conflict of interest.
